# Redetermination of the crystal structure of NaTcO_4_ at 100 and 296 K based on single-crystal X-ray data

**DOI:** 10.1107/S2056989017008362

**Published:** 2017-06-16

**Authors:** Konstantin E. German, Mikhail S. Grigoriev, Bogdan L. Garashchenko, Alexander V. Kopytin, Ekaterina A. Tyupina

**Affiliations:** a A.N. Frumkin Institute of Physical Chemistry and Electrochemistry, Russian Academy of Sciences, 31 Leninsky prospekt, 119071 Moscow, Russian Federation; b Medical University Reaviz, 2 Krasnobogatyrskaya, building 2, 107564 Moscow, Russian Federation; c D. Mendeleyev University of Chemical Technology of Russia, 9 Miusskaya pl., 125047 Moscow, Russian Federation; d Kurnakov Institute of General and Inorganic Chemistry, Russian Academy of Sciences, 31 Leninsky prospect, 119991 Moscow, Russian Federation; e National Research Nuclear University, 31 Kashirskoye sh., 115409 Moscow, Russian Federation

**Keywords:** Technetium, sodium, redetermination, crystal structure, low temperature, high precision

## Abstract

The redetermination of the title compound, sodium pertechnate, from single-crystal CCD data recorded both at 296 and 100 K confirms previous studies based on X-ray powder diffraction film data [Schwochau (1962 ▸). *Z. Naturforsch. Teil A*, **17**, 630; Keller & Kanellakopulos (1963 ▸). *Radiochim. Acta*, **1**, 107–108] and neutron powder diffraction data using the Rietveld method [Weaver *et al.* (2017 ▸). *Inorg. Chem.* **12**, 677–681], but reveals a considerable improvement in precision. The standard uncertainties of the room-temperature structure determination are about seven times lower than those of the neutron diffraction structure determination and about 13 times lower at 100 K, due to the decrease in the amplitude of librations. The crystal expansion could be approximated linearly with a thermal volumic expansion coefficient of 1.19 (12) × 10^−4^ K^−1^. NaTcO_4_ adopts the scheelite (CaWO_4_) structure type in space group type *I*4_1_/*a* with Na and Tc atoms (both with site symmetry -4) replacing Ca and W atoms, respectively.

## Abstract 2

The title compound, NaTcO_4_, forms tetra­gonal crystals both at 100 and 296 K with a thermal volumic expansion coefficient of 1.19 (12) × 10^−4^ K^−1^.

## Chemical context   

Sodium pertechnetate, NaTcO_4_, refers to a group of *d* ^0^-tetroxide anion salts. Since the inception of quantum chemistry, compounds of this type have been models (generally with respect to the MnO_4_^−^ anion) for which the validity of the proposed equations and approximations for the case of *d*-electrons are verified. It was believed that, owing to the *d* ^0^ electronic state, they define the least complex class of compounds of *d*-elements. Such simplicity, due to the absence of *d*-electrons and their pseudospherical symmetry, does by far not imply that any of these compounds show no complex behavior under changing environmental conditions, *e.g.* by changing temperature and/or the strength of the crystal field, and publications on the discovery of a more complex behaviour and properties appeared periodically. For example, for sodium (German *et al.*, 1987*b* ▸, 1993 ▸), potassium (German *et al.*, 1993 ▸; Gafurov & Aliev, 2005 ▸) and caesium (Tarasov *et al.*, 1991 ▸, 1992 ▸) tetra­oxidotechnates, the existence of phase transitions was noted at high temperatures, while for the rhenium analogue, caesium tetra­oxidorhenate, the ability of laser-excited second harmonic generation has been observed (Stefanovich *et al.*, 1991 ▸). Differences for these systems are also observed in the crystal structures. Potassium permanganate crystallizes in the ortho­rhom­bic system (Palenik, 1967 ▸), whereas the per­techne­tate and perrhenate of the same cation crystallize in the tetra­gonal system (Hoppe *et al.*, 1999 ▸; Schwochau, 1962 ▸). Next to the inter­est for the TcO_4_^−^ anion in its sodium salt, sodium cations in general are worth being investigated in detail. For example, sodium salts are known to form hydrates with different hydration numbers and various coordination numbers for the sodium cation. The change in these numbers often occurs in the vitally important temperature range of 309–313 K (German *et al.*, 1987*b* ▸; Tarasov *et al.*, 2015 ▸). Precise structural data of such systems are important for the analyses of transmutation rates in homogeneous systems as noted by Kuo *et al.* (2017 ▸) and in this respect, are more useful than the data of previously determined structures (Kuo *et al.*, 2017 ▸; Ackerman *et al.*, 2016 ▸; German *et al.*, 1987*a* ▸; Spitsyn *et al.*, 1987 ▸; Tarasov *et al.*, 1983 ▸, 1991 ▸). Likewise, Ackerman *et al.* (2016 ▸) have shown that precise structural data are needed for the estimation of the incorporation possibility for ^99^Tc into stable scheelite matrices of different compositions. Another aspect for obtaining more precise structure data on pertechnates is to clarify if pseudo-Jahn–Teller distortions of *d* ^0^-tetra­oxide anions really take place when compared with previous determinations (German *et al.*, 1987*a* ▸; Spitsyn *et al.*, 1987 ▸; Tarasov *et al.*, 1983 ▸, 1991 ▸). In this context we have reinvestigated the crystal structure of NaTcO_4_ that is known from powder diffraction data only, namely by inspection of its X-ray powder diffraction pattern (Schwochau, 1962 ▸; Keller & Kanellakopulos, 1963 ▸) and Rietveld refinement of neutron powder diffraction data (Weaver *et al.*, 2017 ▸).

## Structural commentary   

The structure of anhydrous NaTcO_4_, determined here on the basis of X-ray diffraction data of a single crystal recorded both at room and low temperature, belongs to the CaWO_4_ structural type (space group type *I*4_1_/*a*). The obtained bond lengths and angles are similar to those obtained from previous X-ray powder (Keller & Kanellakopulos, 1963 ▸; Schwochau, 1962 ▸) and neutron powder diffraction studies (Weaver *et al.*, 2017 ▸)

Lattice parameters determined here with the precision of 0.0002-0.0005 Å at 296 K (Table 1 ▸) are close to those of *a* = 5.342 (3) Å, *c* = 11.874 (6) Å given by Weaver *et al.* (2017 ▸). The lattice parameters at 100 K are *a* = 5.2945 (2) Å, *c* = 11.7470 (5) Å (single crystal measurement). These values represent the thermal volumic expansion coefficient of 1.19 (12) × 10 ^−4^ K^−1^. The *c*/*a* ratio in this structure changes from 2.2187 (7) to 2.2223 (4) as a function of the temperature change from 100 to 296 K.

**Figure 1:**
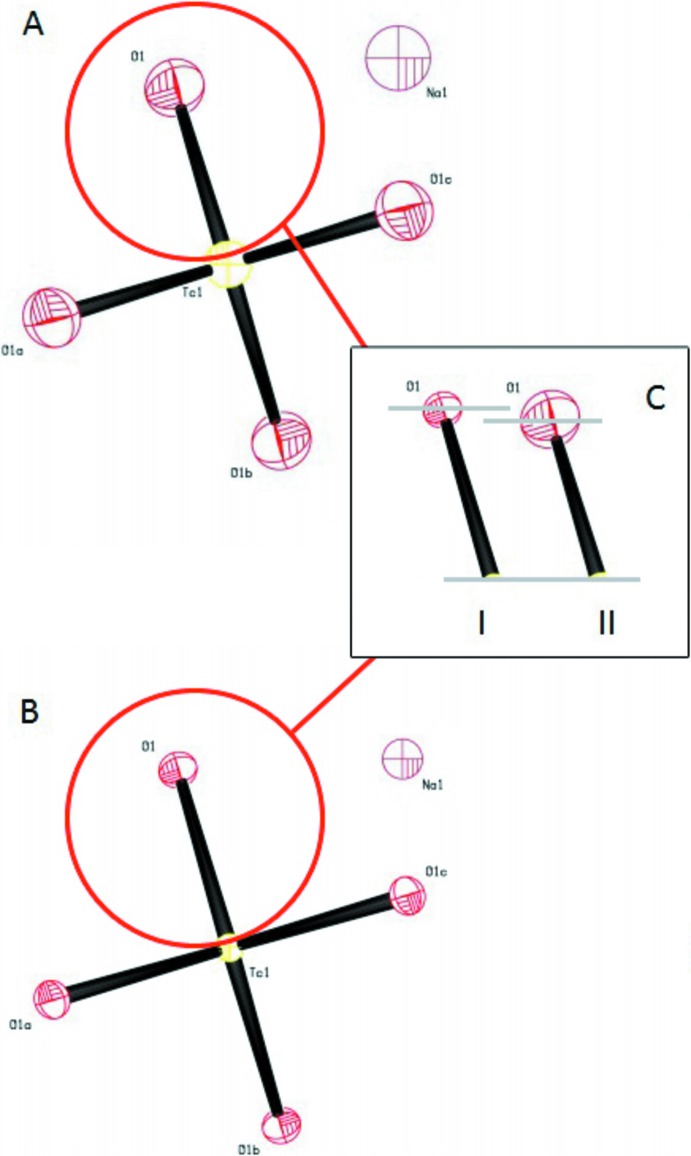
The elongation of bonds upon cooling from 296 K [(II), **A**] to 100 K [(I), **B**] is associated with a decrease in libration (**C**). All displacement ellipsoids are drawn at the 50% probability. [Symmetry codes: (*a*) −*y* + 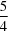, *x* + 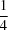, −*z* + 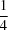; (*b*) −*x* + 1, −*y* + 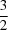, *z*; (*c*) *y* + 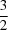, −*x* + 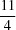., *z* + 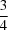.]

Our results confirm that NaTcO_4_ is isostructural to KTcO_4_ and RbTcO_4_ (Keller & Kanellakopulos, 1963 ▸). The structure is composed of three atom types (Na, Tc, O). The Tc and Na atoms occupy special positions with 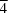 symmetry, Wyckoff positions 4*b* and 4*a*, respectively. The configuration of the TeO_4_^−^ anion is that of a slightly distorted tetra­hedron both at 296 K and at 100 K (Tables 1 ▸ and 2 ▸). The Tc—O distances are 1.7183 (6) Å at 296 K and 1.7208 (3) Å at 100 K. These distances are in good agreement with values known for these ions from the literature (German *et al.*, 1987*a* ▸; Tarasov *et al.*, 1992 ▸; Kuo *et al.*, 2017 ▸; Ackerman *et al.*, 2016 ▸). The elongation of bonds (Fig. 1 ▸), while decreasing the temperature from 296 K to 100 K, can be attributed to a decrease in the libration effect (German *et al.*, 1987*a* ▸). A similar phenomenon has previously been observed in the structure of anilinium pertechnetate (Maruk *et al.*, 2010 ▸).

The greatest distortion of the TcO_4_^−^ anion from an ideal tetra­hedral configuration reported by Weaver *et al.* (2017 ▸) is confirmed by our analysis of the O—Tc—O angles in the NaTcO_4_ structure, but the difference is not as high as in the model from the neutron diffraction experiment (Weaver *et al.*, 2017 ▸). The maximum deviation values are 3.12° at 100 K and 3.08° at 296 K for the sodium salt and are larger in comparison with the potassium and rubidium salts, because the sodium cation has the smallest ionic radius compared to K^+^ and Rb^+^ and hence has the highest polarizing ability. This distortion is insensitive to the temperature change from 100 K to 296 K.

**Figure 2:**
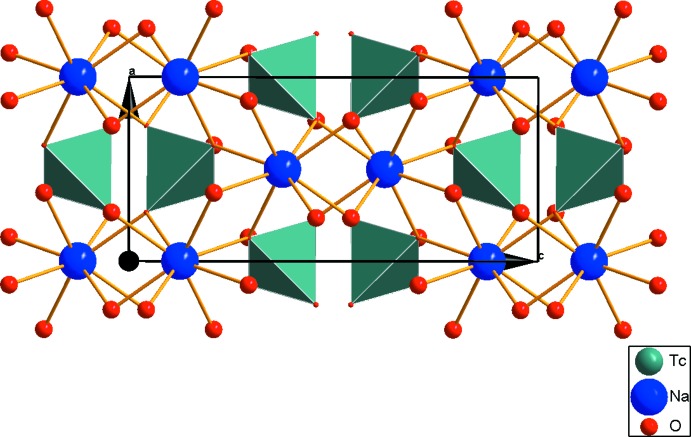
View of the crystal packing of the title compound.

**Figure 3:**
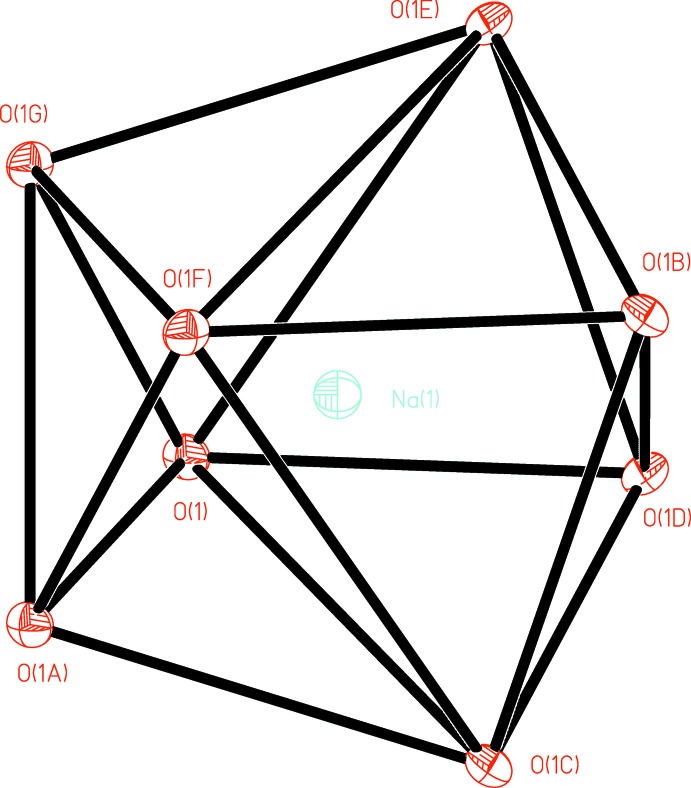
The coordination polyhedron of the sodium cation (data from 100 K measurement).

The packing of Na^+^ cations and TcO_4_^−^ anions in the crystal is presented in Fig. 2 ▸. Each Na^+^ cation is coordinated by eight oxygen atoms that are belonging to four TcO_4_^−^ anions. The resulting coordination polyhedron can be described as a distorted dodeca­hedron (Fig. 3 ▸). The two dihedral angles between pairs of two triangular faces sharing an edge that connects two five-edged vertices of the dodeca­hedron are equal to 21.2 and 30.3°, respectively. The corresponding faces should form an angle of 29.5° for a dodeca­hedron and 0° for a square anti-prism according to the Aslanov–Porai-Koshits criterion (Porai-Koshits & Aslanov, 1972 ▸). Hence the coordi­n­ation polyhedron of the sodium cation is closer to a dodeca­hedron than to a square anti-prism. Each of the four oxygen atoms of an individual TcO_4_^−^ anion is in contact with two sodium cations, so that each TcO_4_^−^ anion is directly contacted with eight sodium cations.

## Synthesis and crystallization   

The synthesis of the title compound was carried out based on neutralization of an aqueous solution of freshly prepared HTcO_4_ with an equivalent qu­antity of 1 *M* aqueous solution of chemically pure sodium hydroxide. The HTcO_4_ solution was made by dissolution of Tc_2_O_7_ sublimed from TcO_2_ in an oxygen flow at 973 K.

## Refinement   

Crystal data, data collection and structure refinement details are summarized in Table 3 ▸. Seven (six) reflections at room (and low) temperature were omitted from refinement due to large differences between observed and calculated intensities.

## Supplementary Material

Crystal structure: contains datablock(s) global, I, II. DOI: 10.1107/S2056989017008362/wm5391sup1.cif

Structure factors: contains datablock(s) I. DOI: 10.1107/S2056989017008362/wm5391Isup2.hkl

Structure factors: contains datablock(s) II. DOI: 10.1107/S2056989017008362/wm5391IIsup3.hkl

Supporting information file. DOI: 10.1107/S2056989017008362/wm5391Isup4.cml

Supporting information file. DOI: 10.1107/S2056989017008362/wm5391IIsup5.cml

CCDC references: 1554512, 1554511

Additional supporting information:  crystallographic information; 3D view; checkCIF report

## Figures and Tables

**Table 1:** Selected geometric parameters (Å, °) at 100 K

Tc1—O1	1.7208 (3)	Na1—O1^ii^	2.5980 (4)
Na1—O1^i^	2.5107 (4)		
			
O1^iii^—Tc1—O1	108.439 (12)	O1^iv^—Tc1—O1	111.56 (3)

Symmetry codes: (i) 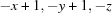; (ii) 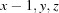; (iii) 
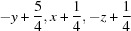
; (iv) 
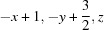
.

**Table 2:** Selected geometric parameters (Å, °) at 296 K

Tc1—O1	1.7183 (6)	O1—Na1^ii^	2.6304 (6)
O1—Na1^i^	2.5357 (6)		
			
O1—Tc1—O1^iii^	111.53 (5)	O1—Tc1—O1^iv^	108.45 (2)

Symmetry codes: (i) 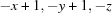; (ii) 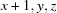; (iii) 
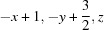
; (iv) 
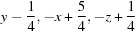
.

**Table 3:** Experimental details

	100 K	296 K
Crystal data		
Chemical formula	NaTcO_4_	NaTcO_4_
*M*_r_	185.9	185.9
Crystal system, space group	Tetragonal, *I*4_1_/*a*	Tetragonal, *I*4_1_/*a*
Temperature (K)	100	296
*a*, *c* (Å)	5.2945 (2), 11.7470 (5)	5.3325 (1), 11.8503 (3)
*V* (Å^3^)	329.29 (3)	336.97 (2)
*Z*	4	4
Radiation type	Mo *K*α	Mo *K*α
μ (mm^−1^)	4.33	4.23
Crystal size (mm)	0.34 × 0.28 × 0.20	0.28 × 0.26 × 0.20

Data collection		
Diffractometer	Bruker Kappa APEXII area-detector	Bruker Kappa APEXII area-detector
Absorption correction	Multi-scan (*SADABS*; Bruker, 2008 ▸)	Multi-scan (*SADABS*; Bruker, 2008 ▸)
*T*_min_, *T*_max_	0.399, 0.478	0.386, 0.485
No. of measured, independent and observed [*I* > 2σ(*I*)] reflections	6909, 678, 661	2371, 365, 350
*R*_int_	0.018	0.016
(sin θ/λ)_max_ (Å^−1^)	0.995	0.805

Refinement		
*R*[*F* ^2^ > 2σ(*F* ^2^)], *wR*(*F* ^2^), *S*	0.009, 0.017, 1.31	0.008, 0.019, 1.16
No. of reflections	678	365
No. of parameters	15	15
Δρ_max_, Δρ_min_ (e Å^−3^)	0.26, −0.34	0.23, −0.33

Computer programs: *APEX2* and *SAINT-Plus* (Bruker, 2008 ▸), *SHELXS2014* (Sheldrick, 2015*a* ▸), *SHELXL2014* (Sheldrick, 2015*b* ▸) and *SHELXTL* (Sheldrick, 2008 ▸).

## supplementary crystallographic information

### (I) Sodium pertechnetate Crystal data

**Table:**

NaTcO_4_	Melting point < 1063 K
*M**_r_* = 185.9	Mo *K*α radiation, λ = 0.71073 Å
Tetragonal, *I*4_1_/*a*	Cell parameters from 5622 reflections
*a* = 5.2945 (2) Å	θ = 4.2–45.4°
*c* = 11.7470 (5) Å	µ = 4.33 mm^−^^1^
*V* = 329.29 (3) Å^3^	*T* = 100 K
*Z* = 4	Fragment, colourless
*F*(000) = 344	0.34 × 0.28 × 0.20 mm
*D*_x_ = 3.750 Mg m^−^^3^	

### (I) Sodium pertechnetate Data collection

**Table:**

Bruker Kappa APEXII area-detector diffractometer	661 reflections with *I* > 2σ(*I*)
ω– and φ–scans	*R*_int_ = 0.018
Absorption correction: multi-scan (*SADABS*; Bruker, 2008)	θ_max_ = 45.0°, θ_min_ = 4.2°
*T*_min_ = 0.399, *T*_max_ = 0.478	*h* = −10→10
6909 measured reflections	*k* = −10→10
678 independent reflections	*l* = −22→23

### (I) Sodium pertechnetate Refinement

**Table:**

Refinement on *F*^2^	0 restraints
Least-squares matrix: full	*w* = 1/[σ^2^(*F*_o_^2^) + (0.0029*P*)^2^ + 0.0769*P*] where *P* = (*F*_o_^2^ + 2*F*_c_^2^)/3
*R*[*F*^2^ > 2σ(*F*^2^)] = 0.009	(Δ/σ)_max_ < 0.001
*wR*(*F*^2^) = 0.017	Δρ_max_ = 0.26 e Å^−^^3^
*S* = 1.31	Δρ_min_ = −0.34 e Å^−^^3^
678 reflections	Extinction correction: SHELXL2014 (Sheldrick, 2015b), Fc^*^=kFc[1+0.001xFc^2^λ^3^/sin(2θ)]^-1/4^
15 parameters	Extinction coefficient: 0.0489 (10)

### (I) Sodium pertechnetate Fractional atomic coordinates and isotropic or equivalent isotropic displacement parameters (Å^2^)

**Table:**

	*x*	*y*	*z*	*U*_iso_*/*U*_eq_	
Tc1	0.5000	0.7500	0.1250	0.00517 (2)	
Na1	0.0000	0.2500	0.1250	0.00980 (7)	
O1	0.73565 (6)	0.62081 (7)	0.04262 (3)	0.00879 (5)	

### (I) Sodium pertechnetate Atomic displacement parameters (Å^2^)

**Table:**

	*U*^11^	*U*^22^	*U*^33^	*U*^12^	*U*^13^	*U*^23^
Tc1	0.00489 (2)	0.00489 (2)	0.00574 (3)	0.000	0.000	0.000
Na1	0.00988 (10)	0.00988 (10)	0.00965 (16)	0.000	0.000	0.000
O1	0.00795 (11)	0.00901 (11)	0.00942 (11)	0.00054 (9)	0.00202 (10)	−0.00104 (10)

### (I) Sodium pertechnetate Geometric parameters (Å, º)

**Table:**

Tc1—O1^i^	1.7208 (3)	Na1—O1^viii^	2.5980 (4)
Tc1—O1^ii^	1.7208 (3)	Na1—O1^ix^	2.5980 (4)
Tc1—O1^iii^	1.7208 (3)	Na1—O1^x^	2.5980 (4)
Tc1—O1	1.7208 (3)	Na1—Na1^xi^	3.9538 (1)
Na1—O1^iv^	2.5107 (4)	Na1—Na1^xii^	3.9538 (1)
Na1—O1^v^	2.5107 (4)	Na1—Na1^xiii^	3.9538 (1)
Na1—O1^vi^	2.5107 (4)	Na1—Na1^xiv^	3.9538 (1)
Na1—O1^vii^	2.5107 (4)	O1—Na1^vii^	2.5107 (4)
Na1—O1^iii^	2.5980 (4)	O1—Na1^xv^	2.5980 (4)
			
O1^i^—Tc1—O1^ii^	108.439 (12)	O1^iii^—Na1—Na1^xi^	129.596 (8)
O1^i^—Tc1—O1^iii^	111.56 (3)	O1^viii^—Na1—Na1^xi^	85.180 (8)
O1^ii^—Tc1—O1^iii^	108.439 (12)	O1^ix^—Na1—Na1^xi^	38.498 (8)
O1^i^—Tc1—O1	108.439 (12)	O1^x^—Na1—Na1^xi^	103.255 (8)
O1^ii^—Tc1—O1	111.56 (3)	O1^iv^—Na1—Na1^xii^	162.891 (8)
O1^iii^—Tc1—O1	108.439 (12)	O1^v^—Na1—Na1^xii^	66.415 (8)
O1^iv^—Na1—O1^v^	127.954 (10)	O1^vi^—Na1—Na1^xii^	40.101 (8)
O1^iv^—Na1—O1^vi^	127.954 (10)	O1^vii^—Na1—Na1^xii^	102.079 (9)
O1^v^—Na1—O1^vi^	76.700 (17)	O1^iii^—Na1—Na1^xii^	38.498 (8)
O1^iv^—Na1—O1^vii^	76.700 (17)	O1^viii^—Na1—Na1^xii^	103.255 (8)
O1^v^—Na1—O1^vii^	127.954 (10)	O1^ix^—Na1—Na1^xii^	85.180 (8)
O1^vi^—Na1—O1^vii^	127.954 (10)	O1^x^—Na1—Na1^xii^	129.596 (8)
O1^iv^—Na1—O1^iii^	149.332 (14)	Na1^xi^—Na1—Na1^xii^	123.484 (2)
O1^v^—Na1—O1^iii^	67.259 (8)	O1^iv^—Na1—Na1^xiii^	66.415 (8)
O1^vi^—Na1—O1^iii^	78.599 (12)	O1^v^—Na1—Na1^xiii^	102.079 (9)
O1^vii^—Na1—O1^iii^	73.985 (7)	O1^vi^—Na1—Na1^xiii^	162.891 (8)
O1^iv^—Na1—O1^viii^	73.985 (7)	O1^vii^—Na1—Na1^xiii^	40.101 (8)
O1^v^—Na1—O1^viii^	78.599 (12)	O1^iii^—Na1—Na1^xiii^	85.180 (8)
O1^vi^—Na1—O1^viii^	67.259 (8)	O1^viii^—Na1—Na1^xiii^	129.596 (8)
O1^vii^—Na1—O1^viii^	149.332 (14)	O1^ix^—Na1—Na1^xiii^	103.255 (8)
O1^iii^—Na1—O1^viii^	136.261 (16)	O1^x^—Na1—Na1^xiii^	38.498 (8)
O1^iv^—Na1—O1^ix^	78.599 (12)	Na1^xi^—Na1—Na1^xiii^	84.064 (3)
O1^v^—Na1—O1^ix^	149.332 (14)	Na1^xii^—Na1—Na1^xiii^	123.484 (2)
O1^vi^—Na1—O1^ix^	73.985 (7)	O1^iv^—Na1—Na1^xiv^	102.079 (9)
O1^vii^—Na1—O1^ix^	67.259 (8)	O1^v^—Na1—Na1^xiv^	40.101 (8)
O1^iii^—Na1—O1^ix^	97.976 (6)	O1^vi^—Na1—Na1^xiv^	66.415 (8)
O1^viii^—Na1—O1^ix^	97.976 (6)	O1^vii^—Na1—Na1^xiv^	162.891 (8)
O1^iv^—Na1—O1^x^	67.259 (8)	O1^iii^—Na1—Na1^xiv^	103.255 (8)
O1^v^—Na1—O1^x^	73.985 (7)	O1^viii^—Na1—Na1^xiv^	38.498 (8)
O1^vi^—Na1—O1^x^	149.332 (14)	O1^ix^—Na1—Na1^xiv^	129.596 (8)
O1^vii^—Na1—O1^x^	78.599 (12)	O1^x^—Na1—Na1^xiv^	85.180 (8)
O1^iii^—Na1—O1^x^	97.976 (6)	Na1^xi^—Na1—Na1^xiv^	123.484 (2)
O1^viii^—Na1—O1^x^	97.976 (6)	Na1^xii^—Na1—Na1^xiv^	84.064 (4)
O1^ix^—Na1—O1^x^	136.261 (16)	Na1^xiii^—Na1—Na1^xiv^	123.484 (2)
O1^iv^—Na1—Na1^xi^	40.101 (8)	Tc1—O1—Na1^vii^	137.472 (19)
O1^v^—Na1—Na1^xi^	162.891 (8)	Tc1—O1—Na1^xv^	118.781 (18)
O1^vi^—Na1—Na1^xi^	102.079 (9)	Na1^vii^—O1—Na1^xv^	101.402 (12)
O1^vii^—Na1—Na1^xi^	66.415 (8)		

Symmetry codes: (i) −*y*+5/4, *x*+1/4, −*z*+1/4; (ii) −*x*+1, −*y*+3/2, *z*; (iii) *y*−1/4, −*x*+5/4, −*z*+1/4; (iv) *x*−1, *y*−1/2, −*z*; (v) *y*−3/4, −*x*+5/4, *z*+1/4; (vi) −*y*+3/4, *x*−3/4, *z*+1/4; (vii) −*x*+1, −*y*+1, −*z*; (viii) −*y*+1/4, *x*−3/4, −*z*+1/4; (ix) −*x*+1, −*y*+1/2, *z*; (x) *x*−1, *y*, *z*; (xi) −*x*, −*y*, −*z*; (xii) −*x*+1/2, −*y*+1/2, −*z*+1/2; (xiii) −*x*, −*y*+1, −*z*; (xiv) −*x*−1/2, −*y*+1/2, −*z*+1/2; (xv) *x*+1, *y*, *z*.

### (II) Sodium tetraoxidotechnetate(VII) Crystal data

**Table:**

O_4_Tc·Na	*D*_x_ = 3.664 Mg m^−^^3^
*M**_r_* = 185.9	Melting point < 1063 K
Tetragonal, *I*4_1_/*a*	Mo *K*α radiation, λ = 0.71073 Å
Hall symbol: -I 4ad	Cell parameters from 2097 reflections
*a* = 5.3325 (1) Å	θ = 4.2–35.2°
*c* = 11.8503 (3) Å	µ = 4.23 mm^−^^1^
*V* = 336.97 (2) Å^3^	*T* = 296 K
*Z* = 4	Fragment, colourless
*F*(000) = 344	0.28 × 0.26 × 0.20 mm

### (II) Sodium tetraoxidotechnetate(VII) Data collection

**Table:**

Bruker Kappa APEX II area-detector diffractometer	365 independent reflections
Graphite monochromator	350 reflections with *I* > 2σ(*I*)
Detector resolution: 9.091 pixels mm^-1^	*R*_int_ = 0.016
ω– and φ–scans	θ_max_ = 34.9°, θ_min_ = 4.2°
Absorption correction: multi-scan (*SADABS*; Bruker, 2008)	*h* = −8→7
*T*_min_ = 0.386, *T*_max_ = 0.485	*k* = −8→8
2371 measured reflections	*l* = −18→18

### (II) Sodium tetraoxidotechnetate(VII) Refinement

**Table:**

Refinement on *F*^2^	0 restraints
Least-squares matrix: full	*w* = 1/[σ^2^(*F*_o_^2^) + (0.0071*P*)^2^ + 0.0846*P*] where *P* = (*F*_o_^2^ + 2*F*_c_^2^)/3
*R*[*F*^2^ > 2σ(*F*^2^)] = 0.008	(Δ/σ)_max_ < 0.001
*wR*(*F*^2^) = 0.019	Δρ_max_ = 0.23 e Å^−^^3^
*S* = 1.16	Δρ_min_ = −0.33 e Å^−^^3^
365 reflections	Extinction correction: SHELXL, Fc^*^=kFc[1+0.001xFc^2^λ^3^/sin(2θ)]^-1/4^
15 parameters	Extinction coefficient: 0.136 (3)

### (II) Sodium tetraoxidotechnetate(VII) Fractional atomic coordinates and isotropic or equivalent isotropic displacement parameters (Å^2^)

**Table:**

	*x*	*y*	*z*	*U*_iso_*/*U*_eq_	
Tc1	0.5000	0.7500	0.1250	0.01434 (6)	
Na1	0.0000	0.2500	0.1250	0.02546 (16)	
O1	0.73494 (12)	0.62442 (11)	0.04342 (6)	0.02225 (12)	

### (II) Sodium tetraoxidotechnetate(VII) Atomic displacement parameters (Å^2^)

**Table:**

	*U*^11^	*U*^22^	*U*^33^	*U*^12^	*U*^13^	*U*^23^
Tc1	0.01355 (6)	0.01355 (6)	0.01591 (7)	0.000	0.000	0.000
Na1	0.0261 (2)	0.0261 (2)	0.0242 (4)	0.000	0.000	0.000
O1	0.0200 (2)	0.0225 (2)	0.0242 (3)	0.0005 (2)	0.0050 (2)	−0.0030 (2)

### (II) Sodium tetraoxidotechnetate(VII) Geometric parameters (Å, º)

**Table:**

Tc1—O1^i^	1.7183 (6)	Na1—O1^vii^	2.5357 (6)
Tc1—O1	1.7183 (6)	Na1—O1^iii^	2.6303 (6)
Tc1—O1^ii^	1.7183 (6)	Na1—O1^viii^	2.6303 (6)
Tc1—O1^iii^	1.7183 (6)	Na1—O1^ix^	2.6303 (6)
Na1—O1^iv^	2.5357 (6)	Na1—O1^x^	2.6303 (6)
Na1—O1^v^	2.5357 (6)	O1—Na1^vii^	2.5357 (6)
Na1—O1^vi^	2.5357 (6)	O1—Na1^xi^	2.6304 (6)
			
O1^i^—Tc1—O1	108.45 (2)	O1^iii^—Na1—Na1^xii^	129.245 (14)
O1^i^—Tc1—O1^ii^	108.45 (2)	O1^viii^—Na1—Na1^xii^	85.049 (14)
O1—Tc1—O1^ii^	111.53 (5)	O1^ix^—Na1—Na1^xii^	38.652 (13)
O1^i^—Tc1—O1^iii^	111.53 (5)	O1^x^—Na1—Na1^xii^	103.569 (14)
O1—Tc1—O1^iii^	108.45 (2)	O1^iv^—Na1—Na1^xiii^	163.325 (14)
O1^ii^—Tc1—O1^iii^	108.45 (2)	O1^v^—Na1—Na1^xiii^	65.896 (14)
O1^iv^—Na1—O1^v^	128.283 (18)	O1^vi^—Na1—Na1^xiii^	40.383 (14)
O1^iv^—Na1—O1^vi^	128.283 (18)	O1^vii^—Na1—Na1^xiii^	102.250 (15)
O1^v^—Na1—O1^vi^	76.17 (3)	O1^iii^—Na1—Na1^xiii^	38.652 (13)
O1^iv^—Na1—O1^vii^	76.17 (3)	O1^viii^—Na1—Na1^xiii^	103.569 (14)
O1^v^—Na1—O1^vii^	128.283 (18)	O1^ix^—Na1—Na1^xiii^	85.049 (14)
O1^vi^—Na1—O1^vii^	128.283 (18)	O1^x^—Na1—Na1^xiii^	129.245 (14)
O1^iv^—Na1—O1^iii^	148.68 (2)	Na1^xii^—Na1—Na1^xiii^	123.539 (1)
O1^v^—Na1—O1^iii^	67.149 (15)	O1^iv^—Na1—Na1^xiv^	65.896 (14)
O1^vi^—Na1—O1^iii^	79.04 (2)	O1^v^—Na1—Na1^xiv^	102.250 (15)
O1^vii^—Na1—O1^iii^	73.993 (11)	O1^vi^—Na1—Na1^xiv^	163.325 (14)
O1^iv^—Na1—O1^viii^	73.993 (11)	O1^vii^—Na1—Na1^xiv^	40.383 (14)
O1^v^—Na1—O1^viii^	79.04 (2)	O1^iii^—Na1—Na1^xiv^	85.049 (14)
O1^vi^—Na1—O1^viii^	67.149 (15)	O1^viii^—Na1—Na1^xiv^	129.245 (14)
O1^vii^—Na1—O1^viii^	148.68 (2)	O1^ix^—Na1—Na1^xiv^	103.569 (14)
O1^iii^—Na1—O1^viii^	136.88 (3)	O1^x^—Na1—Na1^xiv^	38.652 (13)
O1^iv^—Na1—O1^ix^	79.04 (2)	Na1^xii^—Na1—Na1^xiv^	83.973 (2)
O1^v^—Na1—O1^ix^	148.68 (2)	Na1^xiii^—Na1—Na1^xiv^	123.539 (1)
O1^vi^—Na1—O1^ix^	73.993 (11)	O1^iv^—Na1—Na1^xv^	102.250 (15)
O1^vii^—Na1—O1^ix^	67.149 (15)	O1^v^—Na1—Na1^xv^	40.383 (14)
O1^iii^—Na1—O1^ix^	97.762 (10)	O1^vi^—Na1—Na1^xv^	65.896 (14)
O1^viii^—Na1—O1^ix^	97.762 (10)	O1^vii^—Na1—Na1^xv^	163.325 (14)
O1^iv^—Na1—O1^x^	67.149 (15)	O1^iii^—Na1—Na1^xv^	103.569 (14)
O1^v^—Na1—O1^x^	73.993 (11)	O1^viii^—Na1—Na1^xv^	38.652 (13)
O1^vi^—Na1—O1^x^	148.68 (2)	O1^ix^—Na1—Na1^xv^	129.245 (14)
O1^vii^—Na1—O1^x^	79.04 (2)	O1^x^—Na1—Na1^xv^	85.049 (14)
O1^iii^—Na1—O1^x^	97.762 (10)	Na1^xii^—Na1—Na1^xv^	123.539 (1)
O1^viii^—Na1—O1^x^	97.762 (10)	Na1^xiii^—Na1—Na1^xv^	83.973 (2)
O1^ix^—Na1—O1^x^	136.88 (3)	Na1^xiv^—Na1—Na1^xv^	123.539 (1)
O1^iv^—Na1—Na1^xii^	40.383 (14)	Tc1—O1—Na1^vii^	138.27 (3)
O1^v^—Na1—Na1^xii^	163.325 (14)	Tc1—O1—Na1^xi^	118.74 (3)
O1^vi^—Na1—Na1^xii^	102.250 (15)	Na1^vii^—O1—Na1^xi^	100.96 (2)
O1^vii^—Na1—Na1^xii^	65.896 (14)		

Symmetry codes: (i) −*y*+5/4, *x*+1/4, −*z*+1/4; (ii) −*x*+1, −*y*+3/2, *z*; (iii) *y*−1/4, −*x*+5/4, −*z*+1/4; (iv) *x*−1, *y*−1/2, −*z*; (v) *y*−3/4, −*x*+5/4, *z*+1/4; (vi) −*y*+3/4, *x*−3/4, *z*+1/4; (vii) −*x*+1, −*y*+1, −*z*; (viii) −*y*+1/4, *x*−3/4, −*z*+1/4; (ix) −*x*+1, −*y*+1/2, *z*; (x) *x*−1, *y*, *z*; (xi) *x*+1, *y*, *z*; (xii) −*x*, −*y*, −*z*; (xiii) −*x*+1/2, −*y*+1/2, −*z*+1/2; (xiv) −*x*, −*y*+1, −*z*; (xv) −*x*−1/2, −*y*+1/2, −*z*+1/2.
